# 664. Diagnostic Performance of a Multiplexed Gastrointestinal PCR Panel on Identifying Diarrheal Pathogens in Children Undergoing Hematopoietic Stem Cell Transplant

**DOI:** 10.1093/ofid/ofad500.727

**Published:** 2023-11-27

**Authors:** Yue Tao, Xi Mo, Jing Chen, Yijun XIA

**Affiliations:** Shanghai Children’s Medical Center, Shanghai, Shanghai, Shanghai, China; Pediatric Translational Medicine Institute, Shanghai Children's Medical Center, shangahai, Shanghai, China; Department of Hematology and Oncology, Shanghai Children's Medical Center, shanghai, Shanghai, China; bioMérieux (Shanghai) Company, Limited, Shanghai, Shanghai, China

## Abstract

**Background:**

Diarrhea is a common complication of hematopoietic stem cell transplantation (HSCT) and associated with substantial morbidity, but the etiology is often not identified. The objectives of this study were to evaluate 1) whether the introduce of FilmArray GI panel would increase diagnostic yield, 2) the degree to which pre-transplantation colonization predicts post-transplantation infection.

**Methods:**

From November 2019 and February 2021, a total of 158 patients undergoing HSCT were prospectively included in the study (Figue 1). Stool specimens were obtained from all HSCT recipients prior to conditioning therapy, on 28 ± 7 days after transplantation and at any new episode of diarrhea. All stool samples were tested by FilmArray GI panel, and other clinical microbiological assays were also performed as requested by the treating physician.

**Figure:**
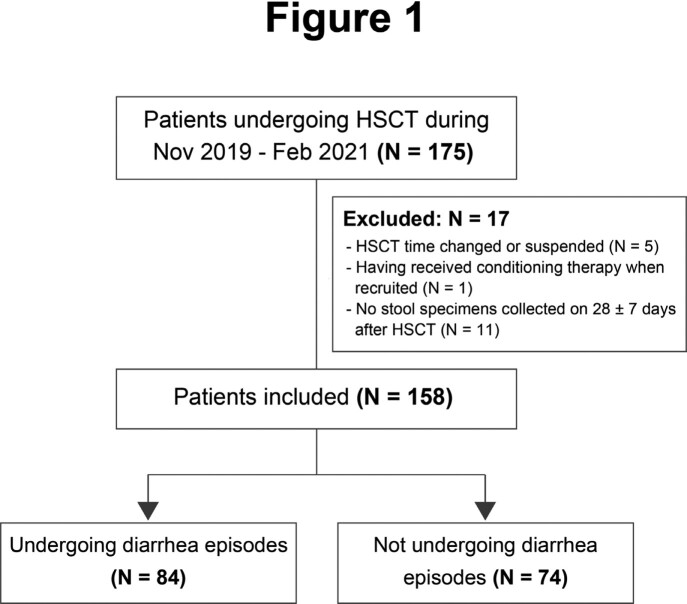

Flowchart of patients’ recruitment. A total of 158 patients were recruited from 175 patients undergoing HSCT during the study period. Eighty-four of the 158 patients underwent diarrhea episodes after transplantation, while the other 74 patients did not.

**Results:**

Eighty-four (53.16%) of the 158 patients developed diarrhea after transplantation. The primary cause of post-transplantation diarrhea was infection (57/84, 67.86%), followed by medication (38/84, 45.24%) and graft-versus-host disease (GVHD, 21/84, 25.00%) (Figure 2). Ninety-five (60.13%) of 158 patients were colonized with at least one gastrointestinal pathogen before conditioning therapy, and the most common colonized pathogens detected were *C. difficile* (62/95, 65.26%), EPEC (21/95, 22.11%), and norovirus (19/95, 20.00%) (Figue 3). The incidence of infectious diarrhea is significantly higher in colonized patients (47/95, 49.47%) than that in non-colonized patients (10/63, 15.87%) (*p* < 0.001) (Figure 4). Pre-transplantation colonization of norovirus was at the highest risk of developing post-transplantation diarrhea (14/19, 73.68%).

**Figure:**
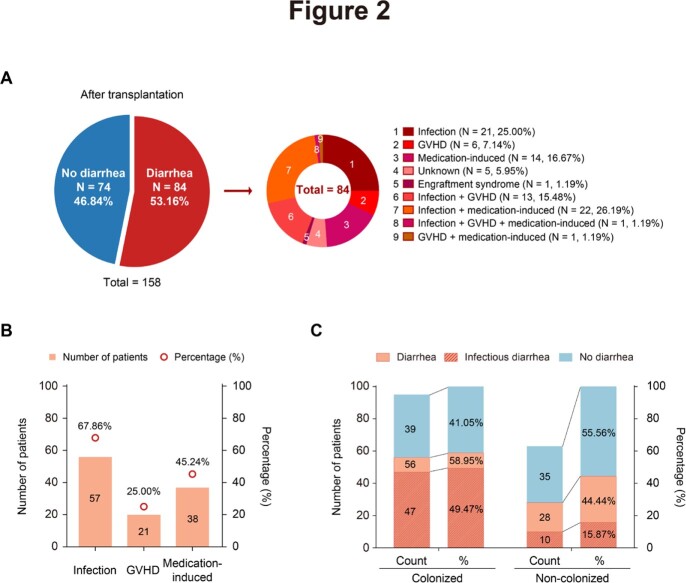

Etiologies of diarrhea after transplantation. (A) Etiologies of diarrhea in 84 patients who underwent diarrhea episodes after transplantation; (B) Etiologies of diarrhea that combined all factors, including infection, GVHD and medication; (C) The incidence of diarrhea and infectious diarrhea in colonized and non-colonized patients.

**Figure:**
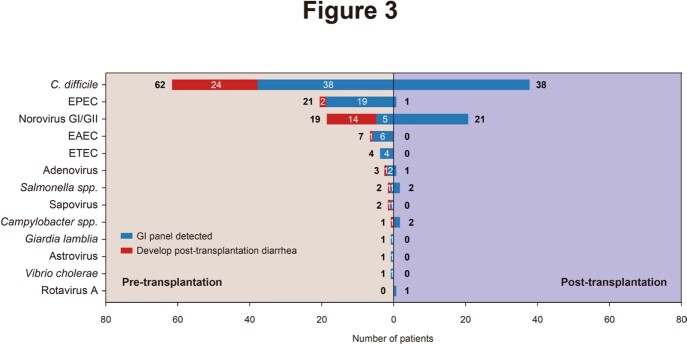

Pathogens detected by FilmArray GI panel in pre- and post- transplantation stool samples. The number of patients who had a clinical relevant post-transplantation diarrheal infection due to pre-transplantation colonization is shown for each pathogen (red part in bar plot).

**Figure:**
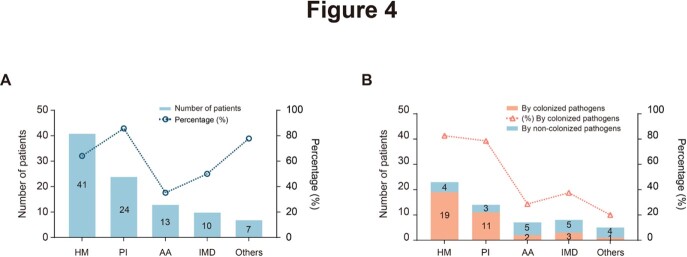

Impact of different underlying diseases on pathogen colonization and subsequent development of infectious diarrhea due to the colonized pathogens. (A) The number and proportions of patients with different underlying diseases who were colonized with gastrointestinal pathogens pre-transplantation. (B) The number and proportions of patients in the colonized group who developed infectious diarrhea due to their colonized pathogens. HM: Hematological malignancies; PI: Primary immunodeficiency; AA: Aplastic anemia; IMD: Inherited metabolic disorders.

**Conclusion:**

FilmArray GI panel can increase the detection rate of diarrheal pathogens in pediatric HSCT patients, and further studies assessing its clinical impact and cost-effectiveness are needed.

**Disclosures:**

**All Authors**: No reported disclosures

